# Clinical Impact of Viscoelastic Testing in Liver Transplantation: A Before-and-After Study of Transfusion Needs and Outcomes

**DOI:** 10.3390/jcm14144882

**Published:** 2025-07-09

**Authors:** Iulian Buzincu, Mihaela Blaj, Eliza Isabela Bărbuță, Adi-Ionuț Ciumanghel, Irina Gîrleanu, Irina Ciumanghel, Ana-Maria Trofin, Vlad Nuțu, Alexandru Năstase, Ramona Cadar, Mihai Zabara, Vlad Carp, Beatrice Cobzaru, Corina Lupascu Ursulescu, Cristian Dumitru Lupașcu

**Affiliations:** 1 Faculty of Medicine, “Grigore T. Popa” University of Medicine and Pharmacy, 700115 Iasi, Romania; buzincu_iulian@yahoo.com (I.B.); miblaj@yahoo.com (M.B.); gilda_iri25@yahoo.com (I.G.); yry_na80@yahoo.com (I.C.); trofin_ana_maria@yahoo.com (A.-M.T.); nutu.vlad@gmail.com (V.N.); alex.gr.nastase@gmail.com (A.N.); tibaramona@yahoo.com (R.C.); zabara_mihai@yahoo.com (M.Z.); corina.ursulescu@umfiasi.ro (C.L.U.); cristian_lupascu@yahoo.com (C.D.L.); 2 Anesthesia and Intensive Care, “St. Spiridon” Emergency Clinical County Hospital, 700111 Iasi, Romania; vlad.sebastian.carp@gmail.com (V.C.); andrabeatrice@yahoo.com (B.C.); 3 Emergency Department, “St. Spiridon” Emergency Clinical County Hospital, 700111 Iasi, Romania; 4 Institute of Gastroenterology and Hepatology, “St. Spiridon” University Hospital, 700111 Iasi, Romania; 5 General Surgery and Liver Transplant Clinic, “St. Spiridon” Emergency Clinical County Hospital, 700111 Iasi, Romania; 6 Department of Radiology, “St. Spiridon” Emergency Clinical County Hospital, 700111 Iasi, Romania

**Keywords:** liver transplantation, viscoelastic testing, transfusion, bleeding

## Abstract

**Background/Objectives**: Liver transplantation (LT) is often complicated by severe bleeding and coagulopathy. Viscoelastic testing (VET) offers real-time, bedside assessment of coagulation and may improve transfusion management compared to standard tests. This study evaluates the clinical impact of VET implementation during liver transplantation on bleeding, transfusion requirements, complications, and mortality in a single Eastern European tertiary transplant center. **Methods**: We conducted a single-center before-and-after study comparing patients undergoing LT before and after the implementation of VET. All procedures were performed by the same surgical and anesthetic team using a standardized protocol. Data were collected retrospectively for the Before VET group and prospectively for the After VET group. We compared transfusion requirements, bleeding, complications, and mortality. **Results**: A total of 59 patients were included, 22 in the After VET group and 37 in the Before VET group. VET implementation was associated with lower intraoperative blood loss (median 4000 mL vs. 6000 mL, *p* = 0.017) and reduced red blood cell (RBC) transfusion volume (670 mL vs. 1000 mL, *p* = 0.008). FFP (0.23 vs. 1.59 units, *p* = 0.007) and platelet use (0.68 vs. 1.81 units, *p* = 0.035) were also significantly lower in the VET group, while fibrinogen use was higher (3.00 g vs. 2.00 g, *p* = 0.036). No differences were observed in complication rates or mortality at 30 days and 1 year in this small before-and-after study. **Conclusions**: VET improved transfusion precision and individualized coagulation management during LT, leading to reduced use of blood products. These findings support the adoption of VET as a standard of care in LT protocols, as it may enhance patient safety, even though no differences in postoperative complications or mortality were observed.

## 1. Introduction

Liver transplantation (LT) is the only life-saving, curative treatment for patients with end-stage liver disease. Massive perioperative bleeding and massive transfusion are often encountered during LT surgery and are important risk factors for both mortality and postoperative complications [1]. Studies report that approximately 30% of patients receive more than 10 units of red blood cells during the procedure [2], and up to 6% may require more than 50 units [3]. The causes of perioperative bleeding are multifactorial. On the one hand, it may result from the complexity of the surgical procedure and technical challenges; on the other hand, it is associated with coagulopathy, either preexisting or acquired intraoperatively [4]. During LT surgery, pre-existing coagulopathy is aggravated by numerous factors such as large-volume blood loss and dilution of coagulation factors, massive transfusion and citrate intoxication, early fibrinolysis, the presence of heparin and heparin-like substances, hypothermia, and hypocalcemia [5,6]. Additionally, large-volume plasma transfusions may contribute to transfusion-associated circulatory overload (TACO) and exacerbate portal hypertension, further increasing bleeding risk [7]. Currently, one of the most difficult intraoperative challenges for anesthesia and surgical teams is represented by finding the balance between preventing bleeding and avoiding thrombotic complications [8].

Although viscoelastic tests (VETs) are increasingly used and widely available in many liver transplantation centers—particularly in the United States [9]—and recent studies have demonstrated that VET-guided perioperative bleeding management significantly reduces transfusion requirements [10,11,12,13,14], VET has not yet been universally adopted as the standard of care in liver transplantation. This is due to variability in transfusion protocols, limited formal training [9], and lower implementation rates in some Eastern European centers. Managing coagulopathy during LT with VET, through thromboelastography (TEG) or rotational thromboelastometry (ROTEM), can offer numerous advantages [15]. These tests can be performed at the patient’s bedside and provide real-time information about the entire coagulation process from clot initiation to fibrinolysis [16]. Compared to standard coagulation tests (SCTs), using VET we can guide the administration of hemostatic agents depending on the deficiencies identified in the coagulation process [17]. Theoretically, we can reduce the administration of unnecessary transfusions of blood, platelets, fibrinogen, or fresh frozen plasma (FFP) and reduce the risk of both hemorrhagic and thrombotic complications, improving the outcome of LT patients [18].

Despite these potential advantages, their utility and effectiveness in clinical practice require further research. This study aims to assess the clinical impact of integrating VET into the management of coagulopathy during orthotopic liver transplantation (OLT), specifically evaluating its effects on bleeding, transfusion requirements, postoperative complications, and mortality. While the use of VET in LT has been explored in previous studies, our work contributes additional evidence by examining its structured implementation in a real-life transplant program from Eastern Europe, where standardized VET-guided protocols are not yet routinely applied. Beyond its clinical endpoints, this study also serves as a quality control assessment of the implementation process within our center.

## 2. Materials and Methods

### 2.1. Study Design and Setting

Initially, due to the lack of VET infrastructure, perioperative coagulation management in OLT at our institution relied on clinical assessment and SCT. In February 2023, VET using the ROTEM Sigma (TEM Innovations GmbH, Munich, Germany) device was introduced, allowing for a transition to a VET-guided transfusion strategy.

This study was conducted at “St. Spiridon” Hospital in Iași, the largest university hospital in the Moldova region of Romania, serving as a tertiary referral center with an active LT program. It was designed as a mixed retrospective-prospective before-and-after study. Data from patients transplanted before February 2023 were collected retrospectively (Before VET group), while data from patients managed after ROTEM implementation were collected prospectively (After VET group).

This study was approved by the Ethics Committee of “Sf. Spiridon” Hospital, Iași, Romania (Approval No. 36) and was conducted in accordance with the principles outlined in the Declaration of Helsinki.

### 2.2. Patient Selection

We enrolled all OLT procedures performed using grafts from brain-dead donors at our institution between September 2016 and December 2024. All patients were adults (≥18 years), and none declined participation in this study. One patient requiring preoperative blood product administration was excluded. The surgeries were performed using the same technique, by the same surgeon, in close collaboration with the same anesthetic team.

This study was conducted in the operating room and the intensive care unit (ICU). Patients were closely monitored throughout their hospitalization and subsequently followed up through scheduled postoperative consultations for one year.

All patients, regardless of the study group (Before VET or After VET), underwent standardized anesthetic and surgical management. Anesthetic induction was performed intravenously with propofol, while maintenance was achieved using sevoflurane, fentanyl, and rocuronium. The surgical technique used was the standard one, with retrohepatic clamping of the inferior vena cava. Hemodynamic optimization was performed using the transpulmonary thermodilution method with the VolumeView/EV1000™ system (Edwards Lifesciences, Irvine, CA, USA). Thus, all patients received standardized perioperative care, with the only difference between groups being the method used for coagulation management (Before VET group vs. After VET group).

### 2.3. The Practical Guide for Coagulopathy Management Used in Our Institution

Patient temperature was maintained above 35 °C using external warming methods and heated infusions at 43 °C via the Level 1™ Fluid Warmer. Ionized calcium and pH levels were actively managed to remain within normal ranges. When not contraindicated, a cell-saver autotransfusion system was used, with autologous transfusion being preferred over allogeneic RBC transfusion to maintain hemoglobin levels above 8 g/dL. Both groups received antifibrinolytic therapy with tranexamic acid (1 g at the beginning of surgery and 1 g at the onset of the neohepatic phase) according to our institutional local protocol. Standard coagulation tests, including fibrinogen measurement and complete blood count, were processed in the hospital laboratory, with results available in approximately one hour. For VET, we used the ROTEM Sigma device, which provided real-time results. Any coagulation test was repeated whenever the patient exhibited diffuse bleeding, at the request of the lead anesthesiologist.

Prophylactic hemostatic blood products or components were avoided in both groups and were administered only if the patient was actively bleeding. When available in the hospital stock, synthetic hemostatic products were preferred. The viscoelastic-guided transfusion strategy implemented in the After VET group was based on clinical recommendations and evidence-based algorithms for ROTEM (A5)-guided bleeding management in liver transplantation, as described by Görlinger et al. [19]. Table 1 summarizes the objective criteria for the administration of hemostatic blood products or components.

### 2.4. Primary and Secondary Outcomes

The primary outcome measured was patient survival at 30 days and one year.

The secondary outcomes included:

- Estimated intraoperative blood loss—measured in milliliters using the cell-saver device, aspirator suction volume, and visual estimation of blood loss from surgical fields and soaked gauzes.
- Intraoperative transfusion requirements:
  ○ the volume of autologous blood (ml) collected and reinfused using the cell-saver;
  ○ the number of allogeneic RBC units and the total volume of allogeneic RBC transfused (ml);
  ○ the number of units of FFP, prothrombin complex concentrate, platelets, and cryoprecipitate transfused;
  ○ the number of patients requiring each of these blood products;
  ○ the total amount of fibrinogen concentrate administered, measured in grams.
- Massive hemorrhage defined as blood loss exceeding one circulating blood volume (CBV) on the day of surgery. CBV was calculated using the formula CBV = 70 mL × ideal body weight (kg), for all patients.
- The occurrence of medical and surgical complications within the first year after transplantation.

Medical complications included:

- major neurological complications, such as altered neurological status, seizures, cerebrovascular complications, and central pontine myelinolysis [20];
- non-infectious respiratory complications, including pleural effusion, pulmonary edema/oedema, atelectasis, and acute respiratory distress syndrome (ARDS) [21];
- cardiovascular complications, including arrhythmia, heart failure, myocardial infarction, and thromboembolism [22];
- acute kidney injury, defined according to the KDIGO criteria [23];
- requirement for Continuous Renal Replacement Therapy (CRRT);
- infectious complications.

Surgical complications, as defined by Agostini et al. [24], included:

- postoperative bleeding;
- thrombosis of large vessels;
- biliary complications;
- surgical reintervention.

### 2.5. Statistical Analysis

All statistical analyses were performed using Jamovi ver. 2.4 (2023). Generative AI tools (ChatGPT, version GPT-4o, developed by OpenAI) were used to improve the clarity and fluency of the English language and to refine the scientific phrasing of the manuscript. All content, including study design, methodology, statistical analysis, results, interpretation of the results, and conclusions, is entirely the original work of the authors. Continuous variables were tested for normality using the Shapiro–Wilk test. Normally distributed continuous variables were presented as mean ± standard deviation (SD) and compared using the independent *t*-test. Non-normally distributed continuous variables were expressed as median (interquartile range, IQR) and compared using the Mann–Whitney U test. To improve data interpretation and visibility, in some cases, the mean was reported alongside the median, even when data were non-normally distributed. Categorical variables were summarized as absolute numbers (*n*) and percentages (%) and compared using the chi-square test or Fisher’s exact test, as appropriate. A *p*-value < 0.05 was considered statistically significant.

## 3. Results

### 3.1. Patient Characteristics

A total of 59 patients who underwent OLT were included in this study, with 22 patients (37.3%) in the After VET group and 37 patients (62.7%) in the Before VET group. The baseline characteristics of the two groups were comparable, with no significant differences in age (*p* = 0.31), sex distribution (*p* = 0.32), weight (*p* = 0.94), height (*p* = 0.42), or BMI (*p* = 0.66).

Regarding preoperative comorbidities, the prevalence of neurological conditions (*p* = 0.76), chronic kidney disease (CKD, *p* = 1), and diabetes mellitus (*p* = 0.19) was similar between groups. However, cardiovascular comorbidities were more frequent in the After VET group (59.1%) compared to the Before VET group (35.1%), though the difference did not reach statistical significance (*p* = 0.07). Similarly, there was a trend towards a higher prevalence of respiratory comorbidities in the After VET group (29.7%) compared to the Before VET group (9.1%), though this difference was not statistically significant (*p* = 0.1).

The most common etiology of liver cirrhosis was hepatitis B + D (37.3%), followed by alcoholic liver disease (27.1%) and hepatitis C (16.9%). The distribution was similar between groups, except for hepatitis C prevalence, which was higher in the Before VET group (24.3%) compared to the After VET group (4.5%) (*p* = 0.07). The proportion of hepatocellular carcinoma was similar in both groups (*p* = 0.18).

Pre-transplant laboratory values, including hemoglobin (*p* = 0.51), platelet count (*p* = 0.16), creatinine (*p* = 0.20), and albumin levels (*p* = 0.12), were comparable between groups. However, INR was significantly higher in the After VET group compared to the Before VET group (1.63 vs. 1.52, *p* = 0.04), suggesting a more pronounced preoperative coagulopathy in patients who receive VET-guided transfusion management.

Intraoperative data, including surgical duration (median 480 min in both groups, *p* = 0.70), cold ischemia time (*p* = 0.66), and warm ischemia time (*p* = 0.11), were not significantly different between the two groups. A detailed comparison of patient characteristics, including demographic data, comorbidities, liver disease etiology, and preoperative laboratory values, as well as intraoperative data such as surgical duration and cold and warm ischemia times, are summarized in Table 2.

### 3.2. Intraoperative Blood Loss and Transfusion Requirements

The estimated intraoperative blood loss was lower in the After VET group compared to the Before VET group, with a median of 4000 mL (IQR: 2925) vs. 6000 mL (IQR: 6200), *p* = 0.017. Similarly, the number of allogeneic RBC units transfused was reduced in the After VET group, with a median of 3 units (IQR: 1.75) vs. 4 units (IQR: 3), *p* = 0.021, while the volume of allogeneic RBC transfused was 670 mL (IQR: 413) vs. 1000 mL (IQR: 810), *p* = 0.008.

Regarding the transfusion of hemostatic products, the mean volume of FFP administered was 0.23 ± 0.75 units in the After VET group compared to 1.59 ± 3.06 units in the Before VET group, *p* = 0.007. The mean number of platelet units transfused was also lower in the After VET group (0.68 ± 1.55 vs. 1.81 ± 2.23, *p* = 0.035).

The mean dose of fibrinogen concentrate administered was 3.00 ± 1.66 g in the After VET group and 2.00 ± 1.37 g in the Before VET group, *p* = 0.036. No statistically significant differences were observed in the use of PCC (*p* = 0.44) or cryoprecipitate (*p* = 0.643) between the two groups.

A detailed summary of these findings can be found in Table 3.

### 3.3. Proportion of Patients Receiving Blood Products and Hemostatic Components

Massive intraoperative hemorrhage occurred in 31.8% of patients in the After VET group compared to 59.5% in the Before VET group (*p* = 0.04, OR = 0.318, 95% CI: 0.1–0.9). The proportion of patients receiving ≥5 units of RBC was 9.1% in the After VET group versus 37.8% in the Before VET group (*p* = 0.018, OR = 0.16, 95% CI: 0.03–0.8). The need for at least one unit of FFP was lower in the After VET group (9.1% vs. 40.5%, *p* = 0.016, OR = 0.147, 95% CI: 0.02–0.7). Similarly, the proportion of patients requiring platelet transfusions (≥1 unit) was lower in the After VET group (18.2% vs. 45.9%, *p* = 0.04, OR = 0.26, 95% CI: 0.07–0.92). There was no statistically significant difference in the administration of PCC (≥500 units) between groups (*p* = 0.51, OR = 0.57, 95% CI: 0.13–2.4), nor in the use of cryoprecipitate (≥1 unit) (*p* = 0.71, OR = 1.42, 95% CI: 0.33–5.98). The proportion of patients receiving ≥3 g of fibrinogen concentrate was higher in the After VET group (50.0% vs. 40.5%, *p* = 0.48, OR = 1.37, 95% CI: 0.5–4.2), although the difference did not reach statistical significance.

**Figure 1:**
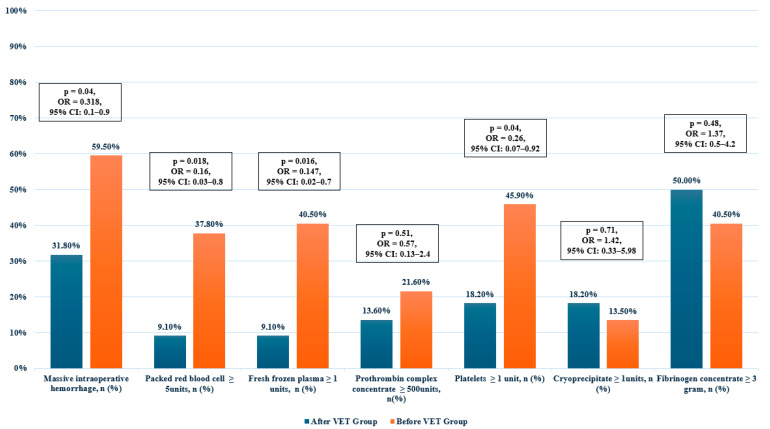
Proportion of patients with massive hemorrhage and those receiving each type of transfusion product, presented with corresponding odds ratios and 95% confidence intervals.

These results are summarized in Table 4 and graphically represented in Figure 1.

### 3.4. Incidence of Postoperative Complications and Mortality in Patients Managed Before and After the Implementation of VET in OLT

The impact of VET implementation on postoperative complications and mortality at one year was analyzed to determine whether VET improved patient outcomes. These data are presented in Table 5. Overall, the incidence of both medical and surgical complications did not differ significantly between the two groups.

Among medical complications, the most frequent were acute kidney injury (AKI) (50.8%), infectious complications (50.8%), and major neurological complications (28.8%). While the incidence of major neurological complications was higher in the After VET group (40.9%) compared to the Before VET group (21.6%), this difference did not reach statistical significance (*p* = 0.11). Similarly, the incidence of non-infectious respiratory complications was lower in the After VET group (22.7% vs. 40.5%, *p* = 0.16), but this difference was also not statistically significant.

For surgical complications, posttransplant bleeding occurred less frequently in the After VET group (13.6%) compared to the Before VET group (27.0%), although the difference was not statistically significant (*p* = 0.33). Likewise, a lower proportion of patients in the After VET group required surgical reintervention (9.1% vs. 27.0%, *p* = 0.17) and developed biliary complications (4.5% vs. 18.9%, *p* = 0.23).

When analyzing the overall rate of complications, there was no statistically significant difference between groups (72.7% in the After VET group vs. 78.4% in the Before VET group, *p* = 0.86).

In terms of mortality outcomes, there was no notable difference between groups at 30 days (9.1% vs. 8.1%, *p* = 1.0) or 1 year (13.6% vs. 16.2%, *p* = 1.0).

## 4. Discussion

In our study, we aimed to determine whether the implementation of VET in the intraoperative management of OLT provided tangible clinical benefits to patients and, if so, to identify these benefits. Our results demonstrate that VET implementation was associated with a significant reduction in massive hemorrhagic episodes, decreased blood loss, and lower transfusion requirements for RBC, FFP, and platelets while leading to an increased use of fibrinogen concentrate. These findings strongly suggest that VET facilitated a more targeted and individualized approach to coagulopathy management, allowing for more precise correction of specific coagulation deficits. Given these improvements in transfusion practices and hemostasis, we expected that these effects would translate into a lower incidence of medical and surgical complications, as well as a reduction in mortality. However, despite the observed advantages in intraoperative blood management, no significant differences were found in the overall complication rates or survival outcomes—results that should be interpreted in the context of this study’s limited sample size.

Our results align with those of Bonnet et al., who also reported that VET use in OLT reduces FFP transfusion while increasing fibrinogen concentrate administration [25]. Similarly, we observed higher fibrinogen use in the VET group, likely reflecting its essential role in hemostasis, especially in ESLD patients where fibrinogen is often low and dysfunctional [26]. This targeted approach, as previously noted by Del Pietri et al., may have contributed to reduced overall transfusion requirements, reinforcing the importance of maintaining adequate fibrinogen levels during OLT [27]. A notable reduction in FFP administration was observed following the introduction of viscoelastic testing, consistent with the findings reported by Scarlatescu et al. [13] and Goerlinger et al. [28]. This finding can be explained by the complex nature of coagulopathy in cirrhotic patients, in whom both procoagulant and anticoagulant factors are reduced [29,30]. Standard coagulation tests such as PT and INR only reflect the deficiency of procoagulant factors and fail to account for the parallel reduction in anticoagulant components, thus offering an incomplete picture of the coagulation status [31]. Relying solely on these parameters may have led to an overestimation of bleeding risk [15] and, consequently, to an overuse of plasma in the Before VET group. This is particularly relevant given that INR is known to have no predictive value for bleeding or blood loss in patients with ESLD [29,32]. Furthermore, a decrease in fibrinogen levels can lead to an artificial prolongation of INR, even when prothrombin activity is preserved [32]. This may result in an overestimation of coagulopathy if INR is used in isolation to guide transfusion decisions. The introduction of VET enabled a more accurate, real-time assessment of global hemostatic function, allowing us to better identify patients who truly required coagulation support, to avoid unnecessary plasma transfusions, and to administer targeted therapy with fibrinogen concentrate when indicated [33].

It has been known since 2009 that platelet transfusion during liver transplantation is associated with decreased one-year survival, primarily due to an increased risk of acute lung injury [34]. Our findings are consistent with previous studies showing that VET management reduces the need for platelet transfusion without increasing the risk of bleeding, [28,35,36] thereby potentially enhancing patient safety and long-term outcomes such as one-year mortality. A lower incidence of non-infectious respiratory complications was noted in the VET group, which may reflect a reduced risk of transfusion-related acute lung injury (TRALI) or TACO, likely due to the decreased exposure to allogeneic blood products.

A trend toward reduced posttransplant bleeding and fewer surgical reinterventions was observed in the VET group, suggesting improved intraoperative hemostatic control. Although these differences did not reach statistical significance, they may indicate a potential clinical benefit in the context of complex procedures such as LT. In our cohort, the total volume of RBC transfused was significantly lower in the VET group. This observation is in line with the findings of Aceto et al., who demonstrated in a meta-analysis that the use of VET during LT is associated with a significant reduction in RBC transfusion [14]. Furthermore, similar to the study by Zamper et al., we did not observe statistically significant differences in the overall complication rate or mortality, despite a favorable trend in the VET group [11].

We believe that, in addition to conventional strategies such as the use of intraoperative cell salvage, maintaining low central and portal venous pressures, avoiding fluid overload, and preventing acidosis and hypothermia [18,37], VET should be considered a mandatory component of anesthetic management aimed at minimizing blood loss and transfusion requirements in LT. Although VET is increasingly explored in trauma and postpartum hemorrhage, its most consistent and widespread adoption has been in liver transplantation [38,39,40]. Our findings, together with current evidence, support the need for enhanced education and training in VET [9], which is crucial for its consistent implementation as standard of care in liver transplantation.

A key strength of our study is the comparability of the two cohorts analyzed. In fact, the After VET group appeared to have more advanced liver disease (as indicated by a higher MELD score, although not statistically significant) and a more pronounced coagulopathy, as reflected by both a higher INR and lower platelet counts compared to the Before VET group even if only the difference in INR reached statistical significance, The increased use of fibrinogen concentrate in the After VET group highlights the advantage of real-time VET in detecting and correcting fibrinogen deficiencies, which may not have been as precisely managed with standard coagulation tests. This further reinforces the concept that VET enables a more refined and individualized correction of coagulopathy, even in a cohort with a greater baseline coagulation impairment.

Our study has several important limitations. First, the small sample size may have limited the ability to detect statistically significant differences in some complications or mortality outcomes, even if a true clinical effect exists. A larger cohort would provide greater statistical power to assess the impact of VET on long-term postoperative outcomes. Although anesthetic and transfusion management were standardized, and all procedures were performed using the same surgical technique by the same team, the before-and-after study design inherently carries certain biases. Over time, both the anesthesia and surgical teams gained additional clinical experience, which may have influenced intraoperative decision-making and patient management. Despite protocolized care, clinical judgment may have introduced variability in transfusion decisions, potentially affecting the comparability of blood product administration between groups. Another important limitation of our study is the availability of hemostatic agents, which may have influenced transfusion practices. While RBC, FFP, cryoprecipitate, and platelets were always available for LT, there were instances when fibrinogen concentrate or PCC was temporarily unavailable. We were unable to include intensive care unit (ICU) length of stay as an outcome measure. A unique aspect of our institution is that LT patients remain in the same dedicated unit under continuous supervision by the transplant team until hospital discharge.

## 5. Conclusions

While VET is not yet available in all centers and its interpretation requires specialized training, our findings support its implementation across all liver transplant centers and advocate for its integration as a standard component of clinical practice. Its use was associated with greater transfusion efficiency and a more individualized, targeted approach to coagulation management, leading to reduced blood product consumption and improved quality of care. However, due to the retrospective design and limited sample size, these findings should be interpreted with caution. Further research in larger, multicenter studies is warranted to confirm its role in reducing postoperative complications and improving patient survival.

## Figures and Tables

**Table 1:** Summary of transfusion triggers based on SCT and VET.

Parameter	Therapy Based on SCT (Before VET Group)	Therapy Based on VET (After VET Group)
FFP	INR > 2, the patient is actively bleeding, and PCC is not available.	CT-EXTEM > 75 s and A5 FIBTEM ≥ 8 mm and PCC is not available.
PCC	INR > 2 and the patient is actively bleeding.	CT-EXTEM > 75 s and A5 FIBTEM ≥ 8 mm.
Platelets	Administered in cases of active bleeding to maintain a platelet count >50,000/μL.	A5 EXTEM < 25 mm and A5 FIBTEM ≥ 8 mm, or severe platelet dysfunction.
Cryoprecipitate	Administered to maintain a fibrinogen concentration of at least 1.5 g/L when fibrinogen concentrate was not available.	Administered when A5 EXTEM < 25 mm and A5 FIBTEM < 8 mm (target A5 FIBTEM ≥ 10 mm) if fibrinogen concentrate is not available.
Fibrinogen Concentrate	Administered to maintain a fibrinogen concentration of at least 1.5 g/L.	Administered when A5 EXTEM < 25 mm and A5 FIBTEM < 8 mm (target A5 FIBTEM ≥ 10 mm).

SCT, standard coagulation tests; FFP, fresh frozen plasma; INR, international normalized ratio; VET, viscoelastic testing; ROTEM, rotational thrombelastometry; CT-EXTEM, clotting time in EXTEM; A5 EXTEM, clot amplitude 5 min after clotting begins in EXTEM; A5 FIBTEM, clot amplitude at 5 min in FIBTEM; PCC, prothrombin complex concentrate.

**Table 2:** Patients’ characteristics.

Variable	All Patients *n* = 59 (100%)	After VET Group *n* = 22 (37.3%)	Before VET Group *n* = 37 (62.7%)	*p*-Value
Age, years, mean ± SD	47.8 ± 9.4	46.1 ± 8.8	48.7 (9.7)	0.31 *
Male gender, *n* (%)	37 (62.7%)	12 (54.5%)	25 (67.6%)	0.32 ***
Weight, kg, mean ± SD	75.7 ± 14.9	75.5 ±16.8	75.8± 13.9	0.94 *
Height, m, mean ± SD	1.72 (0.1)	1.71 (0.1)	1.73 (0.09)	0.42 *
BMI, kg/m^2^, mean ± SD	25.4 (3.9)	25.7 (3.7)	25.2 (4.1)	0.66 *
Comorbidities				
Neurological, *n* (%)	20 (33.9%)	8 (36.4%)	12 (32.4%)	0.76 ***
Respiratory, *n* (%)	13 (22%)	2 (9.1%)	11 (29.7%)	0.1 ****
Cardiovascular, *n* (%)	26 (44.1%)	13 (59.1%)	13 (35.1%)	0.07 ***
CKD, *n* (%)	2 (3.4%)	1 (4.5%)	1 (2.7%)	1 ****
DM, *n* (%)	11 (18.6%)	6 (27.3%)	5 (13.5%)	0.19 ***
Preoperative portal vein thrombosis, *n* (%)	5 (8.5%)	2 (9.1%)	3 (8.1%)	1 ****
Hepatocarcinoma, *n* (%)	11 (18.6%)	2 (9.1%)	9 (24.3%)	0.18 ****
Liver cirrhosis etiology				
Alcoholic liver disease, *n* (%)	16 (27.1%)	8 (36.4%)	8 (21.6%)	0.22 ***
HCV, *n* (%)	10 (16.9%)	1 (4.5%)	9 (24.3%)	0.07 ****
HVB + D, *n* (%)	22 (37.3%)	9 (40.9%)	13 (35.1%)	0.66 ***
Other, *n* (%)	11 (18.6%)	4 (18.2%)	7 (18.9%)	1 ****
Pre-transplant status				
MELD score, mean ± SD	18.8 ± 5.5	20 ± 6.2	18 ± 5	0.23 *
Hemoglobin (g/dL), mean ± SD	11.6 ± 1.8	11.4 ± 2	11.7 ± 1.7	0.51 *
Platelets x10^5^, median (IQR)	63 (55.5)	51.5 (48.2)	69 (57)	0.16 **
INR, median (IQR)	1.57 (0.4)	1.63 (0.5)	1.52 (0.34)	0.04 **
Creatinine (mg/dL), median (IQR)	0.76 (0.3)	0.75 (0.12)	0.84 (0.4)	0.2 **
Albumin (g/dL), mean ± SD	3.1 (1.1)	2.8 (0.65)	3.36 (1)	0.12 **
Intraoperative data				
Surgery duration (min), median (IQR)	480 (90)	480 (100)	480 (70)	0.7 **
Cold ischemia time (min), median (IQR)	220 (112)	204.5 (114)	220 (108)	0.66 **
Warm ischemia time (min), median (IQR)	55 (13.5)	53.5 (13.8)	56 (15)	0.11 **

BMI, body mass index; CKD, chronic kidney disease; DM, diabetes mellitus; HCV, hepatitis C virus; HBV + D, hepatitis B virus + delta virus co-infection; MELD Score, Model for End-Stage Liver Disease; INR, international normalized ratio; SD, standard deviation; IQR, interquartile range;.* *t*-Student test; ** Mann–Whitney test; *** χ^2^-test; **** Fisher’s exact test.

**Table 3:** Intraoperative blood loss and transfusion requirements in all patients and by study groups (Before and After VET).

Variable	All Patients *n* = 59 (100%)		After VET Group *n* = 22 (37.3%)		Before VET Group *n* = 37 (62.7%)		*p* Value
	Mean ± SD	Median (IQR)	Mean + SD	Median (IQR)	Mean + SD	Median (IQR)	
Estimated blood loss (mL)	6768 ± 4643	5500 (3500)	4995 ± 2883	4000 (2925)	7822 ± 5179	6000 (6200)	0.017 **
Autologous blood, volume (mL)	650 ± 582	550 (740)	504 ± 468	470 (528)	738 ± 630	650 (850)	0.17 **
RBC, units	3.95 ± 2.88	3 (3)	2.82 ± 1.68	3 (1.75)	4.62 ± 3.23	4 (3)	0.021 **
RBC, volume (mL)	945 ± 686	730 (690)	670 ± 413	670 (413)	1116 ± 761	1000 (810)	0.008 **
FFP (units)	1.31 ± 2.59	0 (2)	0.23 ± 0.75	0 (0)	1.59 ± 3.06	0 (3)	0.007 **
PCC (units)	492 ± 1015	0 (0)	295 ± 684	0 (0)	608 ± 1162	0 (0)	0.44 **
Platelets (units)	1.39 ± 2.07	0 (3)	0.68 ± 1.55	0 (0)	1.81 ± 2.23	0 (4)	0.035 **
Cryoprecipitate, (units)	1.1 ± 2.86	0 (0)	1.32 ± 3.17	0 (0)	0.97 ± 2.7	0 (0)	0.643 **
Fibrinogen concentrate, (g)	2.34 ± 1.56	2 (2)	3 ± 1.66	2.5 (2.5)	2 ± 1.37	2 (2)	0.036 **

RBC, red blood cells; FFP, fresh frozen plasma; PCC, prothrombin complex concentrate; ** Mann–Whitney test.

**Table 4:** Proportion of patients with massive hemorrhage and those receiving each type of transfusion product, presented with corresponding odds ratios and 95% confidence intervals.

Variable	All Patients	After VET Group	Before VET Group	*p*-Value, OR, CI
Massive intraoperative hemorrhage, *n* (%)	29 (49.2%)	7 (31.8%)	22 (59.5%)	0.04, 0.318, [0.1–0.9] ***
RBC ≥ 5 units, *n* (%)	16 (27.1%)	2 (9.1%)	14 (37.8%)	0.018, 0.16, [0.03–0.8] ****
FFP ≥ 1 units, *n* (%)	17 (28.8)	2 (9.1%)	15 (40.5%)	0.016, 0.147, [0.02–0.7] ****
PCC ≥ 500 units, *n*(%)	11 (18.6%)	3 (13.6%)	8 (21.6%)	0.51, 0.57, [0.13–2.4] ****
Platelets ≥ 1 unit, *n* (%)	21 (35.6%)	4 (18.2%)	17 (45.9%)	0.04, 0.26, [0.07–0.92] ****
Cryoprecipitate ≥ 1 units, *n* (%)	9 (15.3%)	4 (18.2%)	5 (13.5%)	0.71, 1.42, [0.33–5.98] ****
Fibrinogen concentrate ≥ 3 g, *n* (%)	26 (44.1%)	11 (50%)	15 (40.5)	0.48, 1.37, [0.5–4.2] ***

RBC, red blood cell; FFP, fresh frozen plasma; PCC, prothrombin complex concentrate; *** χ^2^-test; **** Fisher’s exact test.

**Table 5:** Incidence of postoperative complications and mortality.

Variable	All Patients *n* = 59 (100%)	After VET Group *n* = 22 (37.3%)	Before VET Group *n* = 37 (62.7%)	*p*-Value
Medical complications (any), *n* (%)	44 (74.6%)	16 (72.7%)	28 (75.7%)	0.8 ***
Major neurological complications, *n* (%)	17 (28.8%)	9 (40.9%)	8 (21.6%)	0.11 ***
Non-infectious respiratory complications, *n* (%)	20 (33.9%)	5 (22.7%)	15 (40.5%)	0.16 ***
Cardiovascular complications, *n* (%)	12 (20.3%)	3 (13.6%)	9 (24.3%)	0.32 ****
AKI, *n* (%)	30 (50.8%)	10 (45.5%)	20 (54.1%)	0.712 ***
CRRT, *n* (%)	9 (15.3%)	3 (13.6%)	6 (16.2%)	1 ***
Infectious complications, *n* (%)	30 (50.8%)	11 (50.0%)	19 (51.4%)	1 ***
Surgical complications (any), *n* (%)	20 (33.9%)	6 (27.3%)	14 (37.8%)	0.586 ***
Posttransplant bleeding, *n* (%)	13 (22.0%)	3 (13.6%)	10 (27.0%)	0.334 ****
Thrombosis of large vessels, *n* (%)	10 (16.9%)	4 (18.2%)	6 (16.2%)	1 ****
Biliary complications, *n* (%)	8 (13.6%)	1 (4.5%)	7 (18.9%)	0.237 ****
Surgical reintervention, *n* (%)	12 (20.3%)	2 (9.1%)	10 (27.0%)	0.179 ****
Any complications, *n* (%)	45 (76.3%)	16 (72.7%)	29 (78.4%)	0.860 ***
Mortality at 30 days, *n* (%)	5 (8.5%)	2 (9.1%)	3 (8.1%)	1 ****
Mortality at 1 year, *n* (%)	9 (15.3%)	3 (13.6%)	6 (16.2%)	1 ****

AKI, acute kidney injury; CRRT, continuous renal replacement therapy; *** χ^2^-test; **** Fisher’s exact test.

## Data Availability

All relevant data are within the paper. The datasets are available from the corresponding author upon reasonable request.

## Abbreviations

The following abbreviations are used in this manuscript:

LT	Liver transplantation
RBC	Red blood cells
VET	Viscoelastic tests
ROTEM	Rotational thromboelastometry
SCT	Standard coagulation tests
FFP	Fresh frozen plasma
OLT	Orthotopic liver transplantation
ICU	Intensive care unit
PCC	Prothrombin Complex Concentrate
CBV	Circulating blood volume
ARDS	Acute respiratory distress syndrome
CRRT	Continuous renal replacement therapy
INR	International Normalized Ratio
